# Psychiatric disorders and mental health care among incarcerated men: A prerelease cross-sectional study in France

**DOI:** 10.1192/j.eurpsy.2025.2443

**Published:** 2025-04-02

**Authors:** Thomas Fovet, Kevin D’Ovidio, Marion Eck, Imane Benradia, Stéphane Duhem, Camille Lancelevée, Pierre Thomas, Marielle Wathelet, Ali Amad

**Affiliations:** 1 Univ. Lille, Inserm, U1172 – Lille Neuroscience & Cognition, Lille, France; 2 Department of Psychiatry, CHU Lille, Lille, France; 3 Fédération Régionale de Recherche en Psychiatrie et Santé Mentale Hauts-de-France (F2RSM Psy), Saint-André-lez-Lille, France; 4 Pôle de Psychiatrie et de Conduites Addictives en Milieu Pénitentiaire, Centre Hospitalier Gérard Marchant, Toulouse, France; 5 EPSM Lille Métropole, Centre Collaborateur de l’Organisation Mondiale de la Santé pour la recherche et la formation en santé mentale, Lille-Hellemmes, France; 6 Équipe Eceve Inserm UMR 1123, Faculté de Médecine Paris-Diderot, Paris, France; 7 Univ. Lille, Inserm, CHU Lille, CIC1403 – Clinical Investigation Center, Lille, France; 8 Unistra, UMR 7363 – SAGE, Sociétés, Acteurs et Gouvernement en Europe, Strasbourg, France; 9 Agence régionale de santé (ARS) des Hauts-de-France, Lille, France

**Keywords:** dual diagnoses, jail, mental disorders, prerelease, substance use disorders

## Abstract

**Background:**

The mental health of incarcerated individuals is a widely recognized public health issue, but little is known about the mental health status of the incarcerated individuals upon release. This study aimed to measure the prevalence of psychiatric disorders and substance use disorders (SUDs) among incarcerated men scheduled to be released from jail soon.

**Methods:**

We conducted a cross-sectional national survey from September 2020 to September 2022 across 26 jails (selected at random) in France. Each participant was interviewed within 30 days prior to their release *via* a structured questionnaire, including the Mini International Neuropsychiatric Interview.

**Results:**

A total of 579 individuals were included in the analysis (participation rate: 66.2%). The prevalence of mood disorders, anxiety disorders, post-traumatic stress disorder, and psychotic episodes were 30.7% (95% confidence interval [CI]: 27.1%–34.6%), 28.7% (95% CI: 25.1%–32.5%), 11.1% (95% CI: 8.8%–13.9%), and 10.5% (95% CI: 8.3%–13.3%), respectively. Additionally, almost half of the individuals had an SUD, and dual disorders were identified in 21.9% (95% CI: 18.8%–25.5%) of the cases. The analysis of mental health care pathways raised questions about access to certain types of care, such as full-time psychiatric hospitalization while in prison, as well as questions about the continuity of care upon release.

**Conclusions:**

This study shows that the mental health of incarcerated men who are scheduled to be released soon is precarious. Complex mental health problems, particularly dual disorders, are common and require better coordination between mental health care systems in prisons and the community.

## Introduction

The mental health of incarcerated individuals is a concerning public health issue [1]. Many psychiatric disorders are overrepresented among incarcerated individuals compared with the general population [2]. Previous studies have consistently reported high prevalence of major depressive disorder, psychosis, post-traumatic stress disorder (PTSD), and substance use disorders (SUDs) among incarcerated individuals [3, 4]. These high prevalences are exacerbated by factors such as a history of trauma, inadequate access to mental health care, and the inherently stressful conditions of incarceration [5]. The significant weight of comorbidity between serious mental illnesses (SMIs) and SUD in prison has also been emphasized in a recent meta-analysis showing that approximately half of the prison population with nonaffective psychosis or major depressive disorder had a comorbid SUD [6].

Importantly, the impact of incarceration on people’s health does not stop at the prison gates. The health of people released from prison has received an increasing amount of attention, as the immediate post-release period is characterized by a range of negative outcomes, particularly increased mortality rates [7]. A recent meta-analysis revealed a markedly elevated rate of death in the first week after release, with alcohol and other drug poisoning, suicide, and cardiovascular disease being the most common causes of death [8]. This problem is far from negligible, given that more than 30 million people are released from jails and prisons worldwide every year [9].

Despite these major findings, few studies have examined the mental health of incarcerated people in the period immediately prior to their release. Research on this topic has generally focused on factors associated with early mortality or criminal recidivism upon release, often relying on registry-based data [7]. These studies have identified the many negative outcomes faced by people suffering from psychiatric disorders, particularly suicide [10], and the well-known “revolving door” phenomenon [11]. However, this type of study provides only limited information on the mental health of incarcerated individuals who were recently released. Given the considerable contribution of mental health issues to negative post-release outcomes, it is essential to directly explore the health of people who are scheduled for release.

It is also crucial to examine how psychiatric care has been implemented during imprisonment and how continuity of care is planned after release. Research has shown that incarcerated people with severe psychiatric disorders receive inadequate health care during their incarceration and minimal mental health support upon release [12]. Understanding the exact mental health conditions of incarcerated people at the time of release, as well as their care pathways, could help to optimize care during this vulnerable period [13].

The main objective of this study is to measure the prevalence of psychiatric disorders and SUDs among incarcerated people in the period immediately preceding their release from jail. The secondary objective of this study is to describe the mental health care pathway of incarcerated people before imprisonment, during imprisonment and after their release.

## Methods

### Population and sampling

The cross-sectional *Mental Health in the Prerelease Jail Population* (MH-PJP) survey was conducted between September 2020 and September 2022 by the *Fédération Régionale de Recherche en Psychiatrie et Santé Mentale* (Regional Federation for Research in Psychiatry and Mental Health, F2RSM Psy).

The number of subjects to be included was calculated via the Clopper–Pearson method [14]. The psychiatric disorder evaluated by the Mini International Neuropsychiatric Interview (MINI) with lowest expected prevalence was psychotic syndrome, with an estimated prevalence rate of 2.3%. Therefore, the required sample size was 800.

Assuming a participation rate of 30%, the goal was to recruit 2,600 incarcerated individuals. The sample was self-weighted and selected in two stages. First, on January 1, 2019, 26 jails were selected at random, on the basis of a draw weighted on the population of jails, among the 90 French jails with a population of over 100 individuals. Second, in each jail, 100 individuals were selected at random among individuals who met the following inclusion criteria: (i) aged 18 years or older, (ii) sentenced (not on remand), and (iii) had an anticipated date of release from prison of at least 30 days and no more than 24 months after the start of the study. This second draw was carried out by the prison administration (*Administration Pénitentiaire*) on August 27, 2020. A total of 2,426 individuals were randomly selected because the number of individuals meeting the inclusion criteria was less than 100 in some facilities (see Supplementary Figure 1 and Supplementary Table 1 for details).

From September 2020 to September 2022, all the individuals selected at random were met by the investigators within 30 days prior to their release. The individuals were screened for the following exclusion criteria: (1) inability to communicate in the French language, (2) mental or psychological incapacity to participate, and (3) opposition to study participation. An information note was given to the eligible men, and an appointment was made before their release. Ethical approval (IDRCB 2019; 79/19-3) was obtained via the French “*Comité de Protection des Personnes*” (CPP).

### Data collection method

Under strict conditions of confidentiality, each participant was interviewed within the prison medical unit by local and trained interviewers (psychiatrists, psychologists, or nurses). A structured questionnaire was administered to the participants in person. Data were collected on a digital tablet or computer and were stored securely.

### Data collected

Sociodemographic data (age, nationality, marital status, children [and dependent children], educational level, monthly income, legal protective measure for vulnerable adults, financial and material assistance in prison, disability living allowance, religious belief, employment status [before imprisonment and planned on release]; and housing [before incarceration and planned on release]); and self-reported criminal/imprisonment status data (juvenile offense, previous imprisonment, reason for current imprisonment according to the International Classification of Crime for Statistical Purposes nomenclature, length of sentence, disciplinary measures, working activity during incarceration, and use of visiting rooms) were collected from each participant. Age was categorized into four groups (18–29, 30–39, 40–49, and ≥ 50 years old). The participants’ level of education was quantified from 0 (“early childhood education”) to 8 (“doctoral or equivalent level”) based on the UNESCO International Standard Classification of Education. Income was categorized as low (≤ €1000/household per month), medium (€1001–€2000/household per month), or high (> €2000/household per month).

The participants were also interviewed about their use of medication and mental health care before and during imprisonment as well as their plans for medication use and mental health care upon release (consultation with a mental health professional, psychiatric hospitalizations, use of psychotropic drugs [i.e., anxiolytics, antidepressants, antipsychotics, hypnotics], use of opioid agonist treatments [OATs; i.e., methadone or buprenorphine]).

For each subject, the MINI (French version 5.0.0), a standardized psychiatric interview, was used to screen for psychiatric disorders as defined by the 10th version of the International Classification of Diseases. The following psychiatric disorders were assessed: (1) mood disorders, that is, manic episode (lifetime, F30); depressive episode (current [past 2 weeks], F32); recurrent depressive disorder (lifetime, F33); and dysthymia (current [past 2 years], F34.1); (2) anxiety disorders, that is, agoraphobia (current, F40.0), panic disorder (current, F41.0), panic disorder with agoraphobia (current, F40.01), social phobias (current, F40.1), generalized anxiety disorder (GAD, current [past 6 months], F41.1); (3) PTSD (current, F43.1); and (4) psychotic episodes (lifetime or current, isolated or recurrent, F2[X]). Antisocial personality disorder (lifetime, F60.2) and insomnia (current [past month], F51) were also assessed. The following SUDs were assessed: (1) alcohol use disorders (AUD, current [past year] harmful use and dependence, F10.1 and F10.2) and (2) drug use disorders (DUD), excluding alcohol, caffeine, and tobacco (current [past year] harmful use and dependence syndrome, F1[X].1 and F1[X].2). Suicide risk (current [past month] and lifetime) was also screened and rated as low, medium, or high. All the interviewers were trained to conduct the MINI over a 1-day session. At the end of the evaluation, each interviewer completed the Clinical Global Impression Severity Scale (CGI-S) [15]. The CGI-S was used to assess the severity of disorders on a scale of 1 (normal, not at all ill) to 7 (among the most extremely ill patients).

Finally, participants reported the perceived effect of incarceration on mental health on a scale ranging from 0 to 10, with 0 indicating a very negative effect and 10 indicating a very positive effect.

### Statistical analyses

The statistical analyses were conducted using R 4.4.2. The characteristics of the sample and criminal/imprisonment status data were described via numbers and percentage values. Prevalences of mental disorders were calculated as percentage values with 95% confidence intervals (CIs). To estimate the prevalence of dual disorders, the diagnoses were grouped as SMIs (including any mood disorder and any psychotic episode) or SUDs (including AUD and DUD). This study was reported in accordance with the STROBE reporting guidelines for observational studies.

## Results

**Figure 1.**
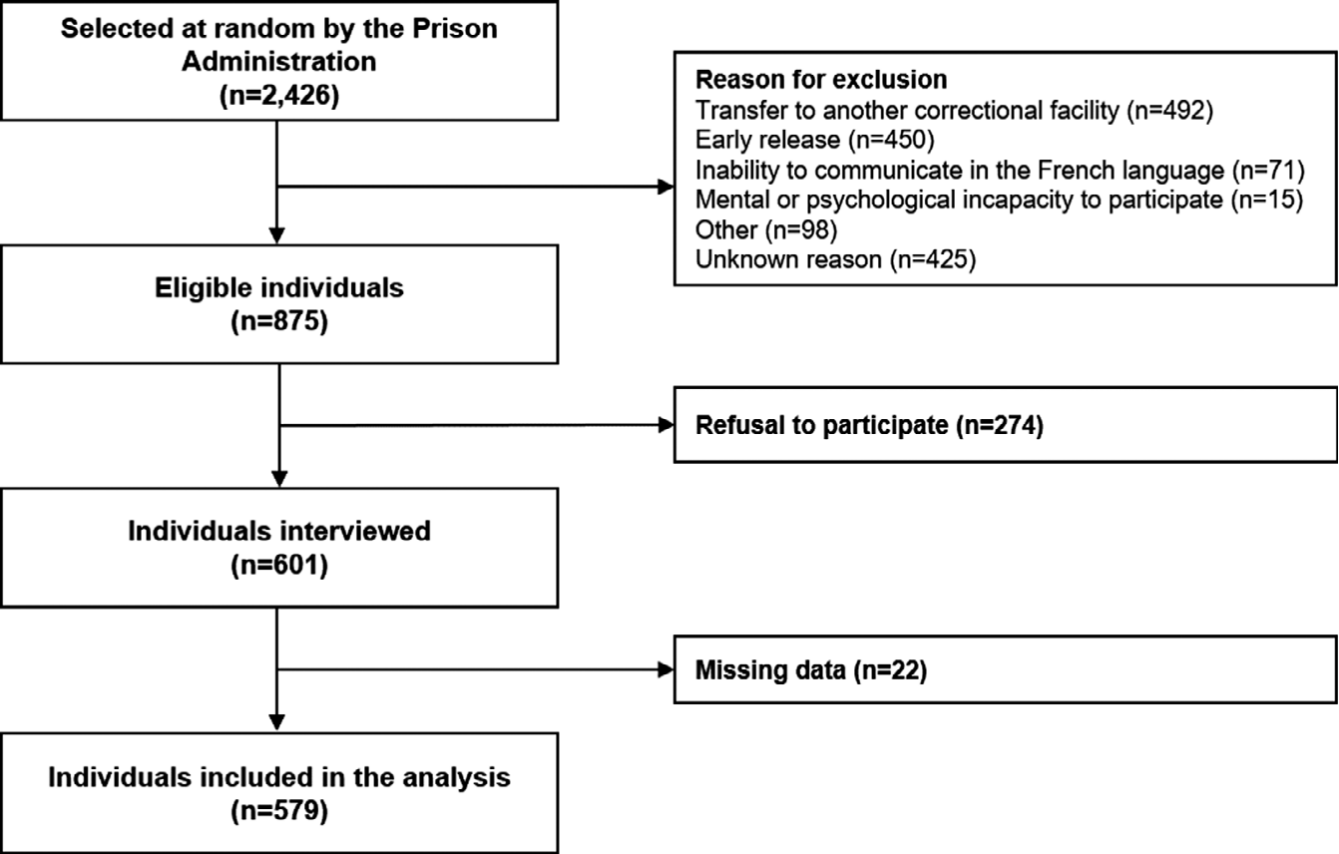
Participation flowchart.

A total of 2,426 men were initially recruited. Among them, 875 were eligible to participate in the study, 601 were ultimately enrolled, and 579 were included for analysis (participation rate: 66.2%). The reasons for noninclusion are detailed in Figure 1.

### Sociodemographic characteristics and criminal/imprisonment status

The sociodemographic characteristics of the interviewees are reported in Table 1. The mean age was 34 years (SD=11.23, min=18, max=89). Our sample consisted mainly of young French men, most of whom were single. Table 2 describes the criminal and imprisonment status of the sample. The most frequent reasons for current incarceration were assault (n=208; 35.9%), robbery/property offenses (n=135; 23.3%), and drug offenses (n=100; 17.3%). More than half of the participants (n=310; 53.5%) were completing sentences of less than a year.

**Table 1.** Sociodemographic characteristics of the sample (n=579 incarcerated men scheduled to be released soon, France, 2021–2022)

		n	%
*Age, years*	18–29	240	41.5%
	30–39	171	29.5%
	40–49	105	18.1%
	≥ 50	62	10.7%
	No response	1	0.2%
*Nationality*	French	435	75.1%
	Other	142	24.5%
	No response	2	0.4%
*Marital status*	Single/cohabiting	472	81.5%
	Married/civil partnership	36	6.2%
	Divorced/separated/widower	71	12.3%
*With child(ren)*	Yes	287	49.6%
	No	285	49.2%
	No response	7	1.2%
*With dependent child(ren)* ^a^	Yes	119	20.6%
	No	447	77.2%
	No response	13	2.2%
*Educational level, ISCED*	0–2	302	52.2%
	3–4	244	42.1%
	≥ 5	21	3.6%
	No response	12	2.1%
*Household legal income*	No income	214	37.0%
	Low (1–1000€/household)	148	25.6%
	Medium (1001–2000€/household)	138	23.8%
	High (>2000€/household)	79	13.6%
*Legal protective measure for vulnerable adults*	Yes	21	3.6%
	No	554	95.7%
	No response	4	0.7%
*Financial and material assistance in prison* ^b^	Yes	230	39.7%
	No	346	59.8%
	No response	3	0.5%
*Disability living allowance*	Yes	65	11.2%
	No	513	88.6%
	No response	1	0.2%
*Religious belief*	Yes	208	35.9%
	No	358	61.8%
	No response	13	2.3%
*Employment before imprisonment*	Yes	253	43.7%
	No (student/housewife/unemployed/retired/undeclared)	326	56.3%
*Planned employment on release*	Yes	115	19.9%
	No (student/housewife/unemployed/retired)	257	44.4%
	Doesn’t know	200	34.5%
	No response	7	1.2%
*Housing before incarceration*	Yes	525	90.7%
	No	53	9.2%
	No response	1	0.2%
*Housing planned on release*	Yes	454	78.4%
	No	32	5.5%
	Doesn’t know	90	15.6%
	No response	3	0.5%

Abbreviation: ISCED, International Standard Classification of Education.

a Dependent children are minors, disabled people or adults attached to the tax household.

b Assistance offered in French prisons to incarcerated people without financial resources.

**Table 2.** Criminal characteristics and imprisonment status of the sample (n=579 incarcerated men scheduled to be released soon, France, 2021–2022)

		n	%
*History of juvenile offending*	Yes	262	45.2%
	No	312	53.9%
	No response	5	0.9%
*Previous imprisonment*	Yes	177	30.5%
	No	401	69.3%
	No response	1	0.2%
*Reason for current imprisonment – ICCS nomenclature*	02-Acts affecting or aimed at affecting a person	208	35.9%
	03-Acts affecting a person of a sexual nature	13	2.2%
	05-Offenses against property without violence or threat	135	23.3%
	06-Acts involving narcotics or other psychoactive substances	100	17.3%
	07-Acts related to fraud, deception, and corruption	6	1.0%
	08-Offenses against public order and state authority	28	4.8%
	09-Offenses against public security and state safety	69	11.9%
	No response	20	3.6%
*Sentence length* ^a^	1–6 months	123	21.2%
	7–12 months	187	32.3%
	>12 months	252	43.5%
	No response	17	3.0%
*Disciplinary measures (solitary confinement, disciplinary transfer)*	Yes	156	26.9%
	No	422	72.9%
	No response	1	0.2%
*Access to working activities*	Yes	298	51.4%
	No	276	47.7%
	No response	5	0.9%
*Use of visiting rooms*	Yes	329	56.8%
	No	245	42.3%
	No response	5	0.9%

Abbreviation: ICCS, International Classification of Crime for Statistical Purposes.

a Time held in jail before the interview.

### Prevalence of psychiatric and SUDs

**Figure 2.**
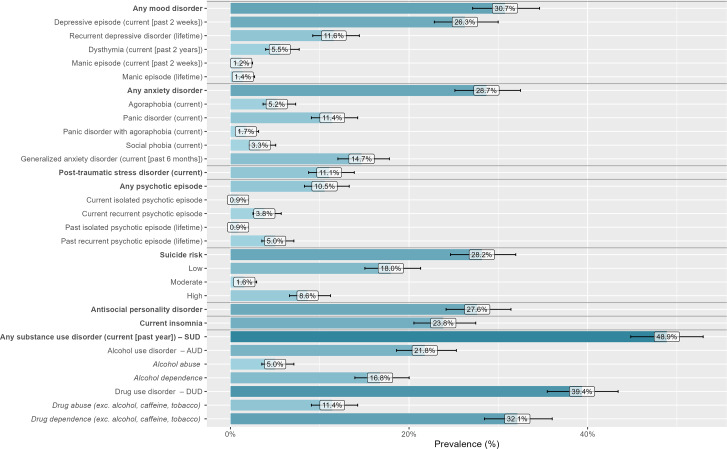
Prevalence of psychiatric disorders and substance use disorders (SUDs) among our sample, according to the Mini International Neuropsychiatric Interview (n=579 incarcerated men soon to be released, France, 2021–2022).

The prevalence of psychiatric disorders and SUDs is reported in Figure 2 (additional details are given in Supplementary Table 2). In total, 66.3% (n=384) of the people interviewed had at least one psychiatric or substance-related disorder (excluding insomnia, suicide risk, and antisocial personality disorder). Nearly half of the sample (46.3%, n=268) had a psychiatric disorder (with or without an SUD), and 20.0% (n=116) had an SUD without a comorbid psychiatric disorder.

**Figure 3.**
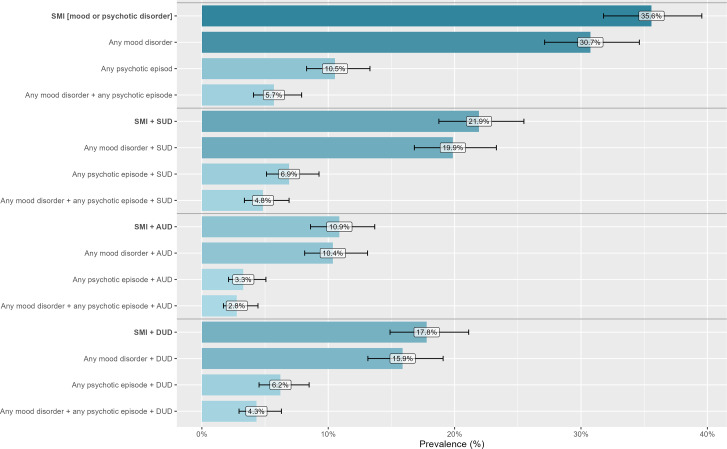
Prevalence of dual diagnoses among our sample, according to the Mini International Neuropsychiatric Interview (n=579 incarcerated men soon to be released, France, 2021–2022).

A total of 127 (21.9%) participants had dual disorders, that is, an SMI (including any mood disorder or any psychotic episode) and an SUD (including AUD and DUD) (see Figure 3; additional details are available in Supplementary Tables 3 and 4).

### Assessment of the severity of disorders (CGI-S)

According to the CGI-S, 39.6% (n=229) of the interviewees were rated as “Normal, not at all ill,” 17.8% (n=103) were rated as “Borderline mentally ill,” 10.4% (n=60) were rated as “Mildly ill,” 16.4% (n=95) were rated as “Moderately ill,” 13.1% (n=76) were rated as “Markedly ill,” 2.4% (n=14) were rated as “Severely ill,” and 0.2% (n=1) were rated as “Among the most extremely ill patients”. Data were missing for one participant.

### Use of medication and mental healthcare services

**Figure 4.**
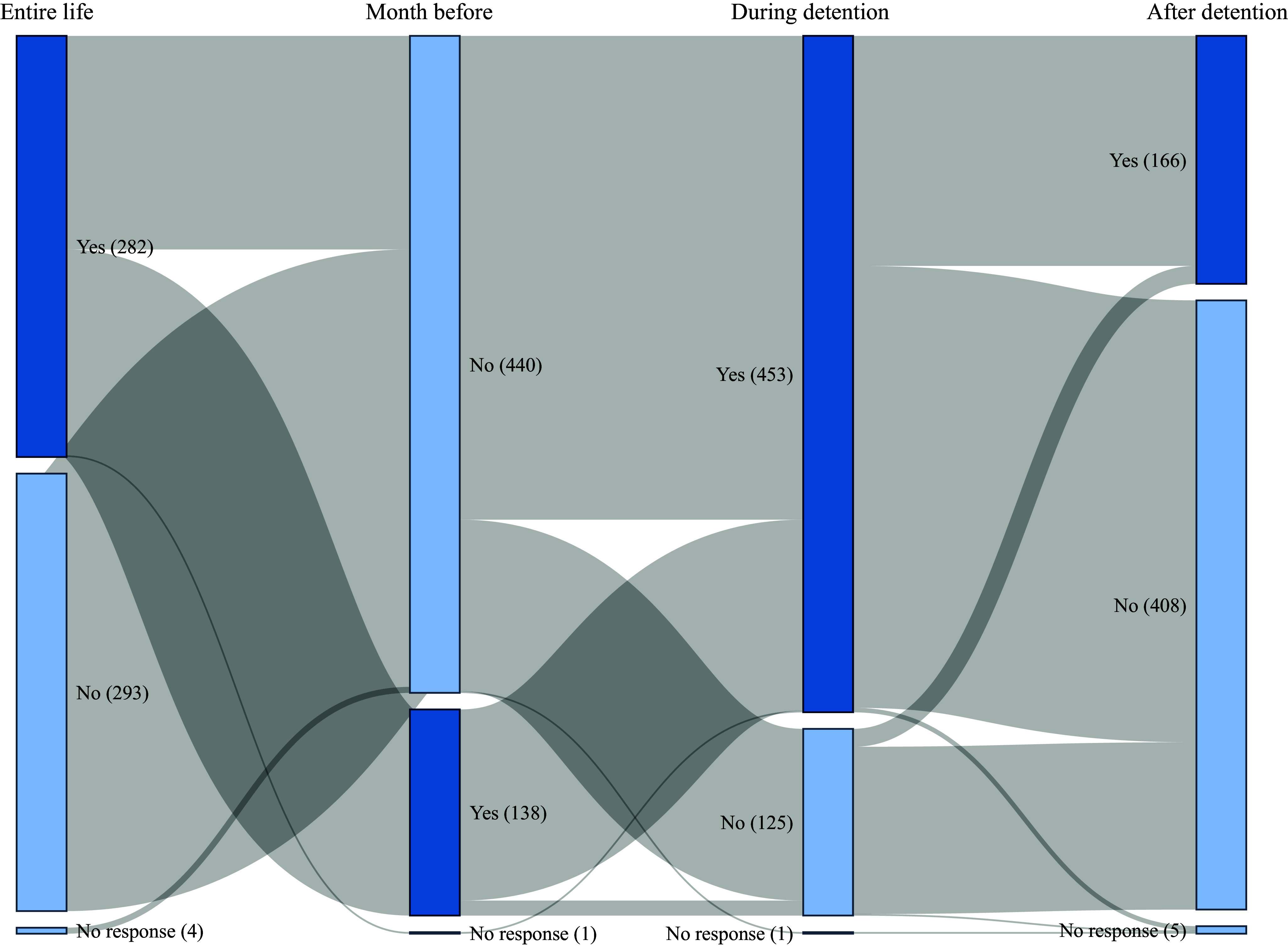
Mental healthcare use (at least one consultation with a mental health professional) before (entire life and month before), during, and planned after imprisonment (n = 579 incarcerated men soon to be released, France, 2021–2022).

Data on the use of outpatient mental healthcare services before and during imprisonment, as well as plans for the use of healthcare upon release are shown in Figure 4. Before imprisonment and in their lifetime, 282 men (48.7%) had at least one consultation with a psychiatrist, psychologist, or addictologist, and 138 men (23.8%) were still being followed up 1 month before imprisonment. Most of our sample (n = 453; 78.2%) had at least one consultation during imprisonment, and 166 men (28.7%) had a consultation scheduled upon release.

Before imprisonment and during their lifetime, 112 men (19.3% of the total sample) had already been hospitalized in psychiatry wards, including 20 men with both voluntary and involuntary hospitalizations (17.9%), 62 men with involuntary hospitalizations (55.3%), and 30 men with voluntary hospitalizations (26.8%). Only 39 men (6.7% of the total sample) were hospitalized in psychiatric wards during imprisonment; 22 (56.4%) men reported that the hospitalizations were voluntary, 5 (12.8%) men reported that the hospitalizations were both voluntary and involuntary, and 12 (30.8%) men reported that the hospitalizations were involuntary.

A total of 320 men (55.3%) received psychotropic drugs (except OAT) during imprisonment. Only 172 men (29.7%) planned to continue treatment upon release. A total of 77 men (13.3%) were treated with OAT during imprisonment, and 62 (10.7%) planned to continue treatment upon release.

A total of 472 men (82.0%) felt that they had satisfactory access to at least one psychiatrist, psychologist, or addictologist during imprisonment (even if they had not used these services themselves). A total of 65 men (11.2%) had taken part in therapeutic activities during imprisonment, and 89 men (15.4%) turned to people other than mental health professionals (cellmates, family members, friends, prison officers, religious figures, etc.) to help with their psychological or psychiatric concerns during imprisonment.

### Subjective effect of incarceration on mental health

The median score was 5, and the mean score was 5.2 (SD=2.9). The mode was 5 (n=143; 24.7%), followed by 8 (n=67; 11.6%), 0 (n=57; 9.8%), and 10 (n=56; 9.8%). Overall, 194 men (33.5%) reported that their incarceration had a negative effect on their mental health (score<5), and 231 (39.9%) reported that it had a positive effect (score>5).

## Discussion

In this study, we found that two-thirds of incarcerated men suffered from a psychiatric disorder or an SUD at the time of their release. The prevalences of mood disorders, anxiety disorders, PTSD, and psychotic episodes were 30.7%, 28.7%, 11.1%, and 10.5%, respectively. Additionally, almost half (48.9%) of the individuals had an SUD, and dual disorders were identified in 21.9% of the cases. The analysis of mental health care pathways raised questions about access to certain types of care, such as full-time psychiatric hospitalization while in prison, as well as questions about the continuity of care upon release.

These results are in line with several previous meta-analyses that have shown a high prevalence of psychiatric disorders among people in prisons [1, 3]. We obtained prevalences that are higher than those reported in international reviews for major depressive disorder (26.3% in our sample vs. 10.2% in Fazel et al. [3]); PTSD (11.1% in our sample vs. 6.2% in Baranyi et al. [16]); and psychosis (10.5% vs. 3.6% in Fazel et al. [3]). The prevalences of AUD and DUD were estimated to be 21.8% and 39.4%, respectively, in our sample versus 24% and 30%, respectively, in an international meta-analysis of 24 studies [4]. Importantly, the prevalence of comorbid SMI and SUD was high in our sample (21.9%), which is in line with a recent meta-analysis that reported a prevalence of 20.7% for co-occurring axis I disorders and SUDs [6].

There are two possible explanations for these particularly high prevalences. First, these rates could be indicative of certain particularities of the situation in French jails. Indeed, the prevalences reported in this study are fairly close to those reported in the most recent national study investigating mental health in French prisons [17]. This survey of 799 incarcerated people sampled at random reported prevalences of 28.6% for mood disorders, 24.0% for anxiety disorders, 9.7% for PTSD, and 17.3% for psychotic disorders. More recently, a study of people entering prisons in northern France reported prevalences of 31.2% for mood disorders, 44.4% for anxiety disorders, 5.0% for PTSD, and 6.9% for psychotic disorders [2]. Therefore, our study highlights the extent to which the incarceration of people suffering from psychiatric disorders remains a widespread problem in France. This situation, which is regularly highlighted by nongovernmental organizations such as *Human Rights Watch* [18], is related not only to the massive referral of people suffering from severe psychiatric disorders to jail and prison in recent decades [19] but also to the dismal conditions of detention perpetuating the poor mental health status of incarcerated people (France has been condemned several times for “inhuman and degrading conditions of detention” by the *European Court of Human Rights*). Our findings, particularly the high prevalence of psychotic disorders among incarcerated individuals, raise important questions about how psychiatric expertise is considered for the assessment of criminal responsibility in France [20]. The second explanation for these findings is related to the methodology of our study. While the majority of epidemiological studies carried out in prisons assess mental health either on entry to prison or during the period of detention, we chose to explore mental health in the 30 days prior to release. Even though the cross-sectional nature of the survey does not allow us to assert that mental health at the time of release can be explained by a deterioration in mental health linked to the conditions of imprisonment, the results do show the precarious state of incarcerated people’s mental health, even a few days before release. Our study also revealed that 33.5% of those surveyed believed that imprisonment had a negative impact on their mental health. The long-term impact of imprisonment on mental health should therefore be the focus of future studies, particularly those using longitudinal designs.

The precarious state of mental health of incarcerated people at the time of their release raises questions about the psychiatric care provided in prisons. Even though most our sample (78.2%) had at least one mental health consultation during imprisonment and 82.0% felt that they had satisfactory access to mental health workers, it seems that the psychiatric care system is struggling to meet the complex care needs of incarcerated people with severe psychiatric disorders. This is reflected in poor access to full-time psychiatric hospitalization (only 6.7% of the sample was admitted to a psychiatric ward during detention, whereas almost 20% had already been admitted to a psychiatric hospital in the community) and therapeutic psychosocial activities (11.2% in our sample). The access to psychiatric hospitalization by incarcerated people has remained problematic in France for many years, and since 2010, the opening of nine full-time inpatient psychiatric wards exclusively for people who are incarcerated has only partially addressed this problem [21].

The provision of psychiatric care after release is important. While 55.3% of the participants reported taking psychotropic medication while in detention, only 29.7% of the sample were planning to continue this treatment. For participants receiving OAT, this proportion decreased from 13.3% during incarceration to 10.7% upon release, despite the fact that the benefits of maintaining OAT upon release have been well documented [22]. Similarly, only 28.7% of the incarcerated people planned to have a psychiatric follow-up. These results could be explained by the dichotomy that exists between mental health and judicial services in France, which sometimes makes it difficult to plan care [23]. Furthermore, coordination between correctional and community health care services is not always optimal: medical centers and psychiatric outpatient facilities are often overloaded, and stigmatization of ex-incarcerated people is not uncommon [24]. These difficulties in accessing mental healthcare are obstacles to reentry into the community after release [25]. These issues are compounded by the social difficulties encountered by people in prison, which are sometimes exacerbated by their incarceration. For example, while 90.7% of the men in our sample had accommodations prior to imprisonment, only 78.4% had housing planned after their release. In terms of employment, only 19.9% had a planned job on release, whereas 43.7% had a job before imprisonment.

Taken together, these factors expose people recently released from prison to numerous risks, including death and recidivism. Overdoses figure prominently in the causes of death, which is consistent with the high prevalence of DUDs in our sample. Suicide is also a major problem among people recently released from prisons. In our study, we identified a high suicide risk (8.6%) among the interviewees. These results should pave the way for concrete action to improve access to mental health care for formerly incarcerated people in the community. The level of evidence for interventions to improve the health of people during imprisonment or in the year after release remains low [26]; however, community reentry programs offer interesting prospects, particularly for substance abuse outcomes [27]. Importantly, these programs address the full range of social and structural issues via individualized support from case managers, which enables the complex mental health needs of this population to be met. Consistent with the results of our studies, our research indicates that the continuity of case worker relationships throughout the prerelease and post-release periods are key factors [27].

This study had several limitations that should be noted. First, with respect to the design of the study, we were only able to interview 23.9% of the 2,426 individuals that were randomly selected. This low rate is essentially because some people were released before their scheduled release date as a result of court decisions. Therefore, in this context, the participation rate was good, as only 274 eligible people out of 875 refused to take part in the survey (refusal rate: 31.3%). Second, we only included sentenced people leaving jails (detention centers before trial or remand centers where incarcerated people on sentences shorter than 2 years reside); therefore, our study did not include incarcerated people released from pretrial detention or sentenced people leaving prisons (detention centers for incarcerated people sentenced to more than 2 years). Further studies are needed to investigate mental health in these facilities. However, it should be noted that jails hold the majority (68%) of incarcerated men in France (49,641 as of 1 November 2022) and 56.4% of sentenced men (30,059 out of 53,227). Third, some limitations of the clinical assessment method should be noted, particularly the fact that these assessments could be carried out only in French, resulting in the exclusion of 71 people who were unable to communicate in French. Additionally, the diagnoses were based on the MINI, and no medical records were available. The validity of the MINI among incarcerated people has already been examined; several recently published studies of mental health among incarcerated people have used the MINI, and it has been validated as a suitable screening tool in prison settings [28]. The MINI has been shown to exhibit good interrater and test–retest reliabilities as well as good convergent validity relative to the Composite International Diagnostic Interview and the Structured Clinical Interview for Diagnostic and Statistical Manual [29]. Fourth, the data on the care pathway and the data on criminal/imprisonment status were self-reported. Future studies should incorporate data from medico-administrative databases and judicial data to gain a better understanding of the barriers to accessing mental health services in this population. Fifth, this study focused exclusively on the health of incarcerated men. Further research is needed to examine the mental health of incarcerated women before their release, as this subgroup faces additional vulnerability factors [30]. Finally, the treatment of individuals diagnosed with mental disorders who have committed crimes varies considerably across countries, reflecting substantial differences in the historical trajectories of criminal justice and psychiatry in each nation [31, 32]. Therefore, caution is required when generalizing these results to countries other than France.

In conclusion, this study revealed that the mental health of incarcerated people who are scheduled for release is precarious. Complex mental health problems, particularly dual disorders, are common and require optimization of the continuity between mental health care in prisons and in the community. These results underscore the need to consider the health of incarcerated individuals as an important part of public health.

## Supporting information

Fovet et al. supplementary material Fovet et al. supplementary material

## Supplementary material

The supplementary material for this article can be found at http://doi.org/10.1192/j.eurpsy.2025.2443.
