# Supplementary material for: Psychiatric disorders and mental health care among incarcerated men: A prerelease cross-sectional study in France
Source: Eur Psychiatry. 2025 Apr 2;68(1):e66. doi: 10.1192/j.eurpsy.2025.2443 (PMC12188331; doi:10.1192/j.eurpsy.2025.2443)
Supplement: Fovet et al. supplementary material [file S0924933825024435sup001.docx]

**Supplementary Material**

**Supplementary Figure 1.** The 26 jails participating in the study

**Supplementary Table 1.** Inclusion rates in the 26 jails participating in the study

**Supplementary Table 2.** Prevalence of psychiatric disorders and substance use disorders (SUD) among our sample, according to the Mini International Neuropsychiatric Interview (n = 579 incarcerated men soon to be released, France, 2021-2022).

**Supplementary Table 3.** Prevalence of dual diagnoses among our sample, according to the Mini International Neuropsychiatric Interview (n = 579 incarcerated men soon to be released, France, 2021-2022).

**Supplementary Table 4.** Prevalence of dual diagnoses among our sample according to the Mini International Neuropsychiatric Interview (n = 579 incarcerated men soon to be released, France, 2021-2022).

*
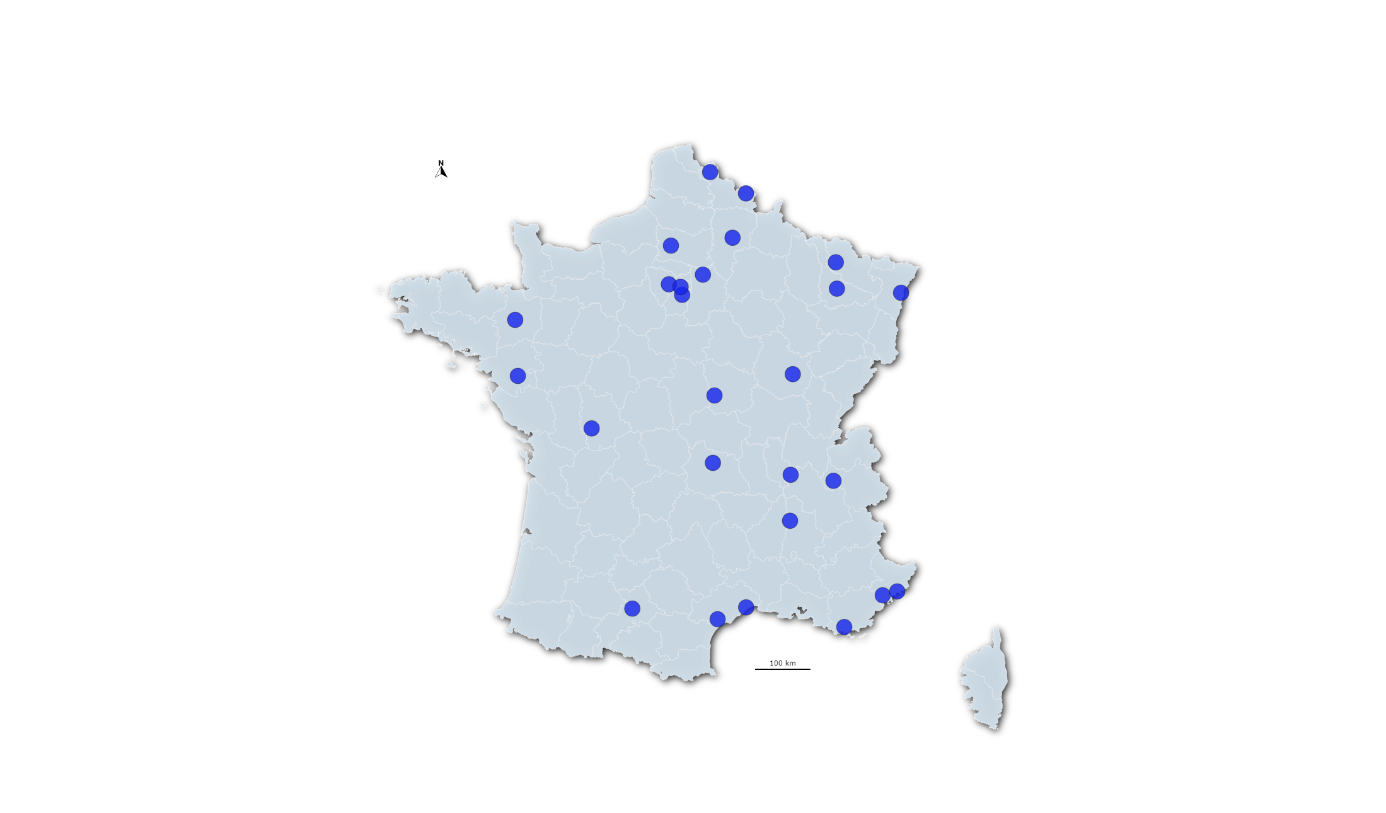
*

**Supplementary Figure 1.** The 26 jails participating in the study

**Supplementary Table 1.** Inclusion rates in the 26 jails participating in the study

| Jail | Number of individuals included | Number of individuals drawn at random | Inclusion rate |
| --- | --- | --- | --- |
| Metz | 43 | 100 | 43,0% |
| Béziers | 39 | 100 | 39,0% |
| La Farlède | 36 | 100 | 36,0% |
| Bois-d’Arcy | 34 | 100 | 34,0% |
| Laon | 31 | 100 | 31,0% |
| Nice | 27 | 100 | 27,0% |
| Maubeuge | 21 | 77 | 27,3% |
| Riom | 27 | 100 | 27,0% |
| Sequedin | 25 | 100 | 25,0% |
| Dijon | 18 | 67 | 26,9% |
| Fleury-Mérogis | 26 | 100 | 26,0% |
| Vivonne | 26 | 100 | 26,0% |
| Villeneuve-lès-Maguelone | 25 | 100 | 25,0% |
| Seysses | 24 | 100 | 24,0% |
| Nantes | 22 | 100 | 22,0% |
| Valence | 21 | 100 | 21,0% |
| Beauvais | 18 | 100 | 18,0% |
| Grasse | 18 | 100 | 18,0% |
| Maxéville | 16 | 100 | 16,0% |
| Fresnes | 15 | 100 | 15,0% |
| Nevers | 6 | 44 | 13,6% |
| Chauconin-Neufmontiers | 15 | 100 | 15,0% |
| Corbas | 15 | 100 | 15,0% |
| Strasbourg | 16 | 100 | 16,0% |
| Vezin-le-Coquet | 11 | 100 | 11,0% |
| Chambéry | 4 | 38 | 10,5% |
| Total | **579** | **2 426** | **23,9%** |

**Supplementary Table 2.** Prevalence of psychiatric disorders and substance use disorders (SUD) among our sample, according to the Mini International Neuropsychiatric Interview (n = 579 incarcerated men soon to be released, France, 2021-2022).

|  | **n** | **%** | **95% CI** |
| --- | --- | --- | --- |
| **Any mood disorder** | **178** | **30.7%** | **[27.1% - 34.6%]** |
| *Depressive episode (current [past 2 weeks])* | 152 | 26.3% | [22.8% - 30.0%] |
| *Recurrent depressive disorder (lifetime)* | 67 | 11.6% | [9.2% - 14.4%] |
| *Dysthymia (current [past 2 years])* | 32 | 5.5% | [3.9% - 7.7%] |
| *Manic episode (current [past 2 weeks])* | 7 | 1.2% | [0.6% - 2.5%] |
| *Manic episode (lifetime)* | 8 | 1.4% | [0.7% - 2.7%] |
| **Any anxiety disorder** | **166** | **28.7%** | **[25.1% - 32.5%]** |
| *Agoraphobia (current)* | 30 | 5.2% | [3.7% - 7.3%] |
| *Panic disorder (current)* | 66 | 11.4% | [9.1% - 14.2%] |
| *Panic disorder with agoraphobia (current)* | 10 | 1.7% | [0.9% - 3.1%] |
| *Social phobia (current)* | 19 | 3.3% | [2.1% - 5.1%] |
| *Generalized anxiety disorder (current [past 6 months])* | 85 | 14.7% | [12.0% - 17.8%] |
| **Post-traumatic stress disorder (current)** | 64 | 11.1% | [8.8% - 13.9%] |
| **Any psychotic episode** | **61** | **10.5%** | **[8.3% - 13.3%]** |
| *Current isolated psychotic episode* | 5 | 0.9% | [0.4% - 2.0%] |
| *Current recurrent psychotic episode* | 22 | 3.8% | [2.5% - 5.7%] |
| *Past isolated psychotic episode (lifetime)* | 5 | 0.9% | [0.4% - 2.0%] |
| *Past recurrent psychotic episode (lifetime)* | 29 | 5.0% | [3.5% - 7.1%] |
| **Any substance use disorder (current [past year]) – SUD** | **283** | **48.9%** | **[44.8% - 52.9%]** |
| Alcohol use disorder – AUD | 126 | 21.8% | [18.6% - 25.3%] |
| *Alcohol abuse* | 29 | 5.0% | [3.5% - 7.1%] |
| *Alcohol dependence* | 97 | 16.8% | [13.9% - 20.0%] |
| Drug use disorder – DUD | 228 | 39.4% | [35.5% - 43.4%] |
| *Drug abuse (exc. alcohol, caffeine, tobacco)* | 66 | 11.4% | [9.1% - 14.2%] |
| *. Amphetamine* | 4 | 0.7% | [0.3% - 1.8%] |
| *. Cannabis* | 16 | 2.8% | [1.7% - 4.4%] |
| *. Cocaine* | 39 | 6.7% | [5.0% - 9.1%] |
| *. Heroin* | 4 | 0.7% | [0.3% - 1.8%] |
| *. Psychotropic drugs* | 4 | 0.7% | [0.3% - 1.8%] |
| *. Others* | 2 | 0.3% | [0.1% - 1.3%] |
| *Drug dependence (exc. alcohol, caffeine, tobacco)* | 186 | 32.1% | [28.4% - 36.0%] |
| *. Amphetamine* | 15 | 2.6% | [1.6% - 4.2%] |
| *. Cannabis* | 46 | 7.9% | [6.0% - 10.4%] |
| *. Cocaine* | 149 | 25.7% | [22.3% - 29.4%] |
| *. Heroin* | 26 | 4.5% | [3.1% - 6.5%] |
| *. Psychotropic drugs* | 28 | 4.8% | [3.4% - 6.9%] |
| *. Others* | 6 | 1.0% | [0.5% - 2.2%] |
| **Antisocial personality disorder** | **160** | **27.6%** | **[24.1% - 31.4%]** |
| **Current insomnia** | **138** | **23.8%** | **[20.5% - 27.5%]** |
| **Suicide risk** | **163** | **28.2%** | **[24.6% - 32.0%]** |
| Low | 104 | 18.0% | [15.0% - 21.3%] |
| Moderate | 9 | 1.6% | [0.8% - 2.9%] |
| High | 50 | 8.6% | [6.6% - 11.2%] |

**Supplementary Table 3.** Prevalence of dual diagnoses among our sample, according to the Mini International Neuropsychiatric Interview (n = 579 incarcerated men soon to be released, France, 2021-2022).

|  | **n** | **%** | **95% CI** |
| --- | --- | --- | --- |
| **SMI [mood or psychotic disorder]** | **206** | **35.6%** | **[31.8% - 39.6%]** |
| Any mood disorder | 178 | 30.7% | [27.1% - 34.6%] |
| Any psychotic episod | 61 | 10.5% | [8.3% - 13.3%] |
| Any mood disorder + any psychotic episode | 33 | 5.7% | [4.1% - 7.9%] |
| **SMI + SUD** | **127** | **21.9%** | **[18.8% - 25.5%]** |
| Any mood disorder + SUD | 115 | 19.9% | [16.8% - 23.3%] |
| Any psychotic episode + SUD | 40 | 6.9% | [5.1% - 9.3%] |
| Any mood disorder + any psychotic episode + SUD | 28 | 4.8% | [3.4% - 6.9%] |
| **SMI + AUD** | **63** | **10.9%** | **[8.6% - 13.7%]** |
| Any mood disorder + AUD | 60 | 10.4% | [8.1% - 13.1%] |
| Any psychotic episode + AUD | 19 | 3.3% | [2.1% - 5.1%] |
| Any mood disorder + any psychotic episode + AUD | 16 | 2.8% | [1.7% - 4.4%] |
| **SMI + DUD** | **103** | **17.8%** | **[14.9% - 21.1%]** |
| Any mood disorder + DUD | 92 | 15.9% | [13.1% - 19.1%] |
| Any psychotic episode + DUD | 36 | 6.2% | [4.5% - 8.5%] |
| Any mood disorder + any psychotic episode + DUD | 25 | 4.3% | [2.9% - 6.3%] |

*Dual diagnoses are defined here as comorbidities between different groups of disorders: 1) severe mental illness (SMI) including any mood disorder and any psychotic episode and 2) SUD including alcohol (AUD) and drugs use disorder (DUD)*

**Supplementary Table 4.** Prevalence of dual diagnoses among our sample according to the Mini International Neuropsychiatric Interview (n = 579 incarcerated men soon to be released, France, 2021-2022).

|  | **n** | **%** |
| --- | --- | --- |
| **SUD + any mood disorder** | **115** | **[16.8% - 23.3%]** |
| SUD + depressive episode (current [past 2 weeks]) | 93 | [13.3% - 19.3%] |
| SUD + recurrent depressive disorder (lifetime) | 41 | [5.3% - 9.5%] |
| SUD + dysthymia | 27 | [3.2% - 6.7%] |
| SUD + Manic episode (current) | 5 | [0.4% - 2.0%] |
| SUD + Manic episode (lifetime) | 8 | [0.7% - 2.7%] |
| **SUD + any anxiety disorder** | **107** | **[15.5% - 21.8%]** |
| SUD + agoraphobia (current) | 19 | [2.1% - 5.1%] |
| SUD + panic disorder (current) | 41 | [5.3% - 9.5%] |
| SUD + panic disorder with agoraphobia (current) | 6 | [0.5% - 2.2%] |
| SUD + social phobia (current) | 15 | [1.6% - 4.2%] |
| SUD + generalized anxiety disorder (current [past 6 months]) | 57 | [7.7% - 12.5%] |
| **SUD + Post-traumatic stress disorder (current)** | **44** | **[5.7% - 10.0%]** |
| **SUD + any psychotic episode** | **40** | **[5.1% - 9.3%]** |
| SUD + current isolated psychotic episode | 5 | [0.4% - 2.0%] |
| SUD + current recurrent psychotic episode | 14 | [1.4% - 4.0%] |
| SUD + past isolated psychotic episode (lifetime) | 3 | [0.2% - 1.5%] |
| SUD + past recurrent psychotic episode (lifetime) | 18 | [2.0% - 4.9%] |
| **AUD + any mood disorder** | **60** | **[8.1% - 13.1%]** |
| AUD + depressive episode (current [past 2 weeks]) | 50 | [6.6% - 11.2%] |
| AUD + recurrent depressive disorder (lifetime) | 20 | [2.2% - 5.3%] |
| AUD + dysthymia | 14 | [1.4% - 4.0%] |
| AUD + Manic episode (current) | 2 | [0.1% - 1.3%] |
| AUD + Manic episode (lifetime) | 6 | [0.5% - 2.2%] |
| **AUD + any anxiety disorder** | **47** | **[6.2% - 10.6%]** |
| AUD + agoraphobia (current) | 7 | [0.6% - 2.5%] |
| AUD + panic disorder (current) | 22 | [2.5% - 5.7%] |
| AUD + panic disorder with agoraphobia (current) | 4 | [0.3% - 1.8%] |
| AUD + social phobia (current) | 8 | [0.7% - 2.7%] |
| AUD + generalized anxiety disorder (current [past 6 months]) | 23 | [2.7% - 5.9%] |
| **AUD + Post-traumatic stress disorder (current)** | **18** | **[2.0% - 4.9%]** |
| **AUD + any psychotic episode** | **19** | **[2.1% - 5.1%]** |
| AUD + current isolated psychotic episode | 2 | [0.1% - 1.3%] |
| AUD + current recurrent psychotic episode | 8 | [0.7% - 2.7%] |
| AUD + past isolated psychotic episode (lifetime) | 2 | [0.1% - 1.3%] |
| AUD + past recurrent psychotic episode (lifetime) | 7 | [0.6% - 2.5%] |
| **DUD + any mood disorder** | **92** | **[13.1% - 19.1%]** |
| DUD + depressive episode (current [past 2 weeks]) | 77 | [10.8% - 16.3%] |
| DUD + recurrent depressive disorder (lifetime) | 33 | [4.1% - 7.9%] |
| DUD + dysthymia | 22 | [2.5% - 5.7%] |
| DUD + Manic episode (current) | 4 | [0.3% - 1.8%] |
| DUD + Manic episode (lifetime) | 4 | [0.3% - 1.8%] |
| **DUD + any anxiety disorder** | **86** | **[12.2% - 18.0%]** |
| DUD + agoraphobia (current) | 17 | [1.8% - 4.7%] |
| DUD + panic disorder (current) | 33 | [4.1% - 7.9%] |
| DUD + panic disorder with agoraphobia (current) | 4 | [0.3% - 1.8%] |
| DUD + social phobia (current) | 11 | [1.1% - 3.4%] |
| DUD + generalized anxiety disorder (current [past 6 months]) | 45 | [5.9% - 10.2%] |
| **DUD + Post-traumatic stress disorder (current)** | **39** | **[5.0% - 9.1%]** |
| **DUD + any psychotic episode** | **36** | **[4.5% - 8.5%]** |
| DUD + current isolated psychotic episode | 4 | [0.3% - 1.8%] |
| DUD + current recurrent psychotic episode | 12 | [1.2% - 3.6%] |
| DUD + past isolated psychotic episode (lifetime) | 3 | [0.2% - 1.5%] |
| DUD + past recurrent psychotic episode (lifetime) | 17 | [1.8% - 4.7%] |
